# Plasticity of host selection by malaria vectors of Papua New Guinea

**DOI:** 10.1186/s13071-017-2038-3

**Published:** 2017-02-21

**Authors:** John B. Keven, Lisa Reimer, Michelle Katusele, Gussy Koimbu, Rebecca Vinit, Naomi Vincent, Edward Thomsen, David R. Foran, Peter A. Zimmerman, Edward D. Walker

**Affiliations:** 1 Papua New Guinea Institute of Medical Research, Vector Borne Diseases Unit, Madang, 511 Madang Papua New Guinea; 2 Department of Microbiology and Molecular Genetics, Michigan State University, 48824 East Lansing, MI USA; 3 Liverpool School of Tropical Medicine and Hygiene, Liverpool, UK; 4 Department of Entomology, Michigan State University, 48824 East Lansing, MI USA; 5 School of Criminal Justice and Department of Integrative Biology, Michigan State University, 48824 East Lansing, MI USA; 6 Center for Global Health and Diseases, Case Western Reserve University, 44106 Cleveland, OH USA

**Keywords:** *Anopheles*, Anthropophilic, Hosts, Malaria, Opportunistic, Selection, Species, Zoophilic

## Abstract

**Background:**

Host selection is an important determinant of vectorial capacity because malaria transmission increases when mosquitoes feed more on humans than non-humans. Host selection also affects the outcome of long-lasting insecticidal nets (LLIN). Despite the recent nationwide implementation of LLIN-based malaria control program in Papua New Guinea (PNG), little is known about the host selection of the local *Anopheles* vectors. This study investigated the host selection of *Anopheles* vectors in PNG.

**Methods:**

Blood-engorged mosquitoes were sampled using the barrier screen method and blood meals analyzed for vertebrate host source with PCR-amplification of the mitochondrial cytochrome *b* gene. Abundance of common hosts was estimated in surveys. The test of homogeneity of proportions and the Manly resource selection ratio were used to determine if hosts were selected in proportion to their abundance.

**Results:**

Two thousand four hundred and forty blood fed *Anopheles* females of seven species were sampled from five villages in Madang, PNG. Of 2,142 samples tested, 2,061 (96.2%) yielded a definitive host source; all were human, pig, or dog. Hosts were not selected in proportion to their abundance, but rather were under-selected or over-selected by the mosquitoes. Four species, *Anopheles farauti* (*sensu stricto*) (*s.s*.), *Anopheles punctulatus* (*s.s*.), *Anopheles farauti* no. 4 and *Anopheles longirostris*, over-selected humans in villages with low LLIN usage, but over-selected pigs in villages with high LLIN usage. *Anopheles koliensis* consistently over-selected humans despite high LLIN usage, and *Anopheles bancroftii* over-selected pigs.

**Conclusions:**

The plasticity of host selection of an *Anopheles* species depends on its opportunistic, anthropophilic or zoophilic behavior, and on the extent of host availability and LLIN usage where the mosquitoes forage for hosts. The high anthropophily of *An. koliensis* increases the likelihood of contacting the LLIN inside houses. This allows its population size to be reduced to levels insufficient to support transmission. In contrast, by feeding on alternative hosts the likelihood of the opportunistic species to contact LLIN is lower, making them difficult to control. By maintaining high population size, the proportion that feed on humans outdoors can sustain residual transmission despite high LLIN usage in the village.

**Electronic supplementary material:**

The online version of this article (doi:10.1186/s13071-017-2038-3) contains supplementary material, which is available to authorized users.

## Background

Host selection is an outcome of the combined effects of a mosquito’s intrinsic (genetic) host preference for a particular host species modulated by extrinsic factors [1, 2]. That is, even though a mosquito may intrinsically prefer a host species due to genetic factors, environmental factors such as availability or accessibility of the preferred host may cause the mosquito to resort to an alternative one. Therefore, host selection is an important determinant of vectorial capacity, because it influences the extent to which mosquitoes in populations feed predominantly on humans or non-humans, [3, 4]. Thus, an *Anopheles* population whose members intrinsically prefer humans are potential vectors of malaria. However, the vectorial capacity of the mosquito population depends on whether extrinsic conditions allow the mosquitoes to feed on humans.

Knowledge of host selection is not only important for evaluating the vectorial capacity of a vector population, but also for guiding vector-based malaria control programs, such as the distribution of long-lasting insecticidal nets (LLIN). The implementation of LLIN is appropriate if we know that local vectors are sufficiently anthropophilic that LLIN will have the intended effect [5]. The inflexibility of anthropophilic species to utilize alternative hosts causes them to pursue humans inside houses and thus increases their likelihood of becoming exposed to the insecticides in the LLIN fabric. The increased likelihood of contacting LLIN enables reduction of their population size to levels insufficient to support transmission. In contrast, if the mosquitoes are opportunistic and exhibit plasticity in host selection, then LLIN may have little effect because these mosquitoes can maintain high population size by feeding on non-human hosts outdoors. By maintaining high population size, the proportion of opportunistic vectors that feed on human individuals before they go under their bed nets can sufficiently sustain residual transmission in the community. Treating the alternative hosts with endectocides lethal to blood-feeding mosquitoes may be more appropriate for controlling such opportunistic vectors. By implementing both methods, the anthropophilic and opportunistic vectors can be successfully controlled.

Human malaria is endemic to Papua New Guinea (PNG) [6]. The main vectors are members of the *Anopheles punctulatus* (*sensu lato*) (*s.l*.) species complex [7, 8], primarily *Anopheles punctulatus* (*sensu stricto*) (*s.s*.), *Anopheles koliensis*, *Anopheles farauti* (*s.s*.) (formerly *Anopheles farauti* no. 1), *Anopheles farauti* no. 4, and *Anopheles hinesorum* (formerly *Anopheles farauti* no. 2) [9–14]. *Anopheles bancroftii* and *Anopheles longirostris* are also vectors of malaria in PNG [12]. Nationwide, an LLIN-based vector control program has been implemented in PNG over the last decade [15–17] to help alleviate the burden of malaria. However, little is known about the host selection behavior of these vectors and their relationship with LLIN usage. This study addresses this knowledge gap and provides guidance on existing as well as new vector control strategies in PNG.

## Methods

### Study sites

**Fig. 1:**
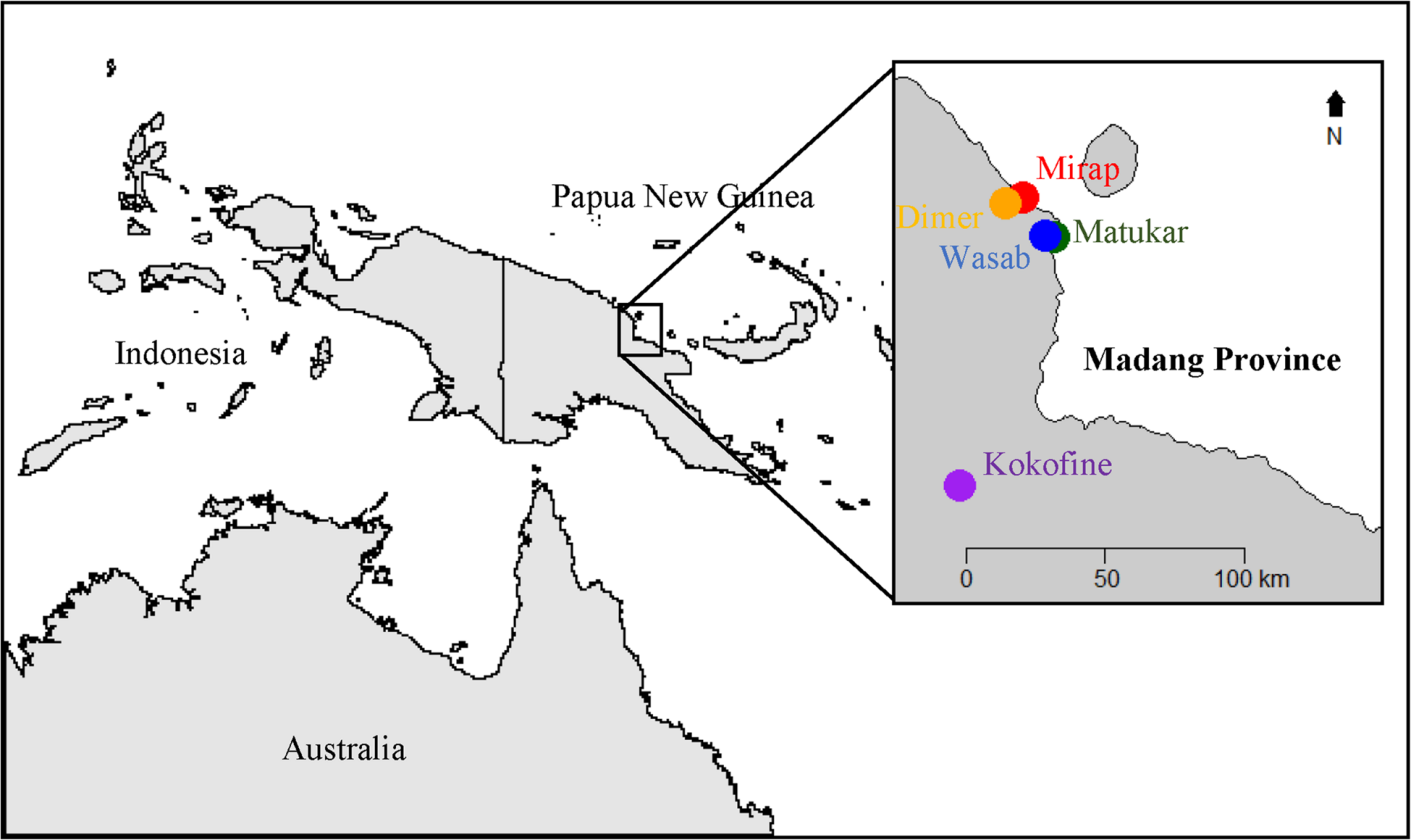
Map of Papua New Guinea showing the five study villages Mirap (*red*), Dimer (*orange*), Matukar (*dark green*), Wasab (*blue*) and Kokofine (*purple*) located in the Madang Province

The study presented here was conducted in five rural villages in Madang Province, PNG (Fig. 1). Four of the villages are located on the north coast of Madang Province. Two of these, Mirap (4°45′67″S, 145°39′59.2″E) and Matukar (4°53′48.9″S, 145°47′04.3″E), sit on a narrow coastal plain, which extends 2–4 km inland before terminating at the foothills of the interior highlands. They are separated by a distance of 22 km, are at an elevation just above sea level, and share similar landscape features of coastal location, secondary forest with brackish swamp, village gardens, and coconut plantations. The two others, Wasab (4°53′28.2″S, 145°45′28.9″E) and Dimer (4°46′33.0″S, 145°37′42.4″E) are located on inland hilltops about 300 m above sea level. Wasab is situated 3 km West of Matukar whilst Dimer is 5.5 km West of Mirap. These inland villages have similar landscape features, consisting of steep-sided, forested hills with streams draining into rivers in nearby valleys. The fifth village, Kokofine (5°41′54.0″S, 145°28′54.0″E), is located on the floodplain of the Ramu River, about 39 km from the nearest coastline. The vegetation consists mainly of lowland swamp and upland secondary forest.

### Demographic survey

A simple household demographic survey was conducted once in each village right at the beginning of the study in 2012. Heads of households were interviewed to gather demographic data that included number of people per household, sex, age, number of LLIN owned, number of people that slept under an LLIN the night before the survey, and number and species of domestic animals owned by the household.

### Mosquito sampling

**Fig. 2:**
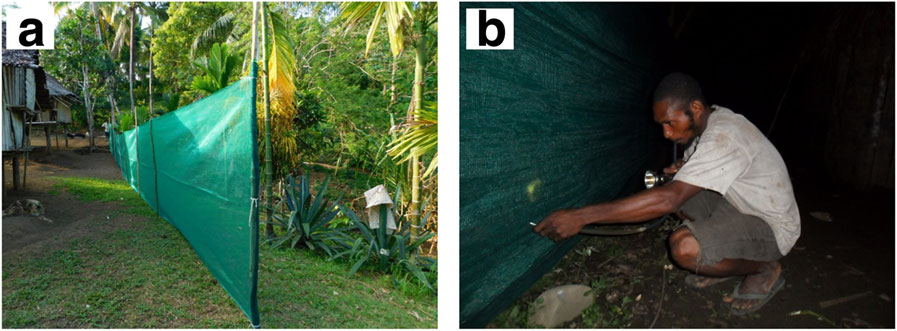
A barrier screen situated at the edge of a hamlet (**a**). Mosquitoes were intercepted on their way into or out of the village and were captured by a trained mosquito collector as they temporarily rested on the surface of the barrier screen (**b**)

Blood-engorged mosquitoes were sampled using the barrier screen (BS) method [18] in the year 2012, 2013 and 2015 with multiple visits to each village. Each visit consisted of 2–6 consecutive nights (see Additional file 1: Table S1 for the exact dates of mosquito sampling). Each BS consisted of a 20 m long, polyethylene shade cloth (70% shading grade) fastened to wooden poles and erected vertically to a height of 2 m (Fig. 2a). Each BS was positioned at locations between the village perimeter and the adjacent bush, with one side facing the bush and the other side facing the village (Fig. 2a). The number of BS per village per night varied from 2 to 10. To reduce sampling biases associated with same sampling location, screens were moved to new locations in each village on consecutive nights. Two trained mosquito collectors were assigned to each BS. One collected from 6:00 pm to midnight before being replaced by the other who continued from midnight to 6:00 am. Collectors sat *c*.20 m away, often in a house with more than one occupants, and visited the BS every 20 min to collect the resting mosquitoes. Mosquitoes were collected using a mouth aspirator with the aid of a hand-held flashlight (Fig. 2b). Captured mosquitoes were placed into a holding container labeled according to the hour of collection. With the aid of a light microscope, non-anophelines and males were separated from female anophelines and blood-engorged female anophelines were identified and separated from the unfed ones. Mosquitoes were kept individually in 1.5 ml microcentrifuge tubes and stored dry on silica gel desiccant at room temperature until processing.

### Mosquito species identification

Female anophelines were identified to species or species groupings based on morphological keys [19, 20]. However, morphological keys are insufficient to allow adequate identification of the species within the *An. punctulatus* (*s.l*.) complex [8]. Therefore, each mosquito identified morphologically as *An. punctulatus*, *An. koliensis*, or *An. farauti* was subjected to polymerase chain reaction (PCR) analyses [8, 21] to identify its true species. DNA template for these reactions was obtained from abdomens of full or partially-fed mosquitoes, extracted using DNeasy Blood & Tissue Kit (Qiagen, Valencia, CA, USA). *An. longirostris* and *An. bancroftii* are each known to consist of several genotypes [22, 23] but whether these genotypes are separate species is still a matter of debate. In this study, their identification was limited to the traditional morphological species.

### Identification of vertebrate host species in mosquito blood meals

To determine the vertebrate species that were fed upon by the mosquitoes, the genomic DNA of each mosquito extracted as noted above was analyzed. A multiplex PCR assay was conducted using a universal reverse primer (UNREV1025 5′-GGT TGT CCT CCA ATT CAT GTT A-3′) and three forward primers targeting a specific region of the mitochondrial cytochrome *b* (*cytb*) gene of three likely hosts: human (*Homo sapiens*), pig (*Sus scrofa*) and dog (*Canis lupus familiaris*), (human741F 5′-GGC TTA CTT CTC TTC ATT CTC TCC T-3′, pig573F 5′-CCT CGC AGC CGT ACA TCT C-3′, and dog368F 5′-GGA ATT GTA CTA TTA TTC GCA ACC AT-3′) [24]. Approximately 20 ng of DNA template of each mosquito was added to a PCR tube (25 μl reaction volume) containing 10 mM Tris at pH 8.3, 50 mM KCl, 1.5 mM MgCl_2_, 0.01% gelatin, 1.0 mM dNTP, 0.5 units of *Taq* polymerase, and 50 pmol of each primer pair. The PCR cycling conditions consisted of one cycle of 95 °C for 5 min (initial denaturation) followed by 35 cycles of 95 °C for 1 min (denaturation), 58 °C for 1 min (annealing), and 72 °C for 1 min (extension), and one cycle of 72 °C for 7 min (final extension). Ten μl of each PCR product was run on an ethidium bromide-stained 2% agarose gel, and visualized using an ultraviolet transilluminator. The host blood source was identified, based on the size of the DNA bands, as human (334 bp), pig (453 bp), or dog (680 bp).

Samples that failed to amplify in the multiplex PCR were subjected to a standard PCR reaction using a generic mammalian primer pair (forward: 5′-CCA TCC AAC ATC TCA GCA TGA TGA AA-3′ and reverse: 5′-GCC CCT CAG AAT GAT ATT TGT CCT CA-3′) which targeted a 395 bp region of the *cytb* gene [25]. The primer pair and approximately 20 ng of a mosquito’s blood meal DNA was added to a 50 μl reaction mixture containing the same reagent concentrations and cycling parameters described for the multiplex PCR above. Samples that failed to amplify with the mammal primer pair were finally tested with a generic avian primer pair (forward: 5′-GAC TGT GAC AAA ATC CCN TTC CA-3′ and reverse: 5′-GGT CTT CAT CTY HGG YTT ACA AGA C-3′) which targeted a 508 bp region of avian *cytb* gene [25] using the same PCR mixture and cycling condition as the mammalian primer pair. Amplicons of the PCR positive samples were purified using QIAquick PCR Purification Kit (Qiagen) and sequenced by direct sequencing. The DNA sequence of each sample was subjected to BLAST search (http://www.ncbi.nlm.nih.gov/blast/Blast.cgi) against vertebrate hosts mitochondrial *cytb* DNA sequences in the GenBank database. Subject sequence that had ≥ 99% sequence similarity to the query sequence was considered the likely host from which the mosquito fed.

### Statistical analyses

Whether the mosquitoes in each of the 5 study villages selected blood hosts in proportion to their relative abundance in the village was determined using two different approaches. Because most hosts utilized by mosquitoes were humans, pigs and dogs (see Results), these analyses were confined to those three hosts. First, a *χ* ^2^ test for homogeneity of proportions was applied on a 3 × 2 frequency table where the rows represent the 3 host species and the 2 columns represent the observed and expected frequencies of blood meals on those hosts. Mosquitoes were considered to have selected hosts disproportionally if the test was statistically significant. Second, the Manly resource selection ratio design II [26] was calculated. It is estimated as the proportion of host *i* of all hosts selected, divided by the proportion of available host *i* of all hosts available to be selected in the community where the sampling was conducted. The ratio equals 1 when host selection is proportional to host availability, greater than 1 when a host is selected greater than its proportionate availability, and less than 1 when a host is selected at less than its proportionate availability. The selection ratio and its 95% confidence interval were calculated using *adehabitat* package in R statistical software (version 3.3.1, R Foundation for Statistical Computing, Vienna, Austria).

The level of LLIN usage for each village was expressed as the proportion of people who reported to have slept under an LLIN the night before the interview. To determine if LLIN affect the success of feeding on human host, logistic regression was used to test whether the probability of feeding on humans versus non-human hosts by a mosquito species was lower in a village with high LLIN usage and higher in a village with lower LLIN usage.

## Results

### *Anopheles* species distribution

A total of 2,440 blood-engorged *Anopheles* mosquitoes, of seven different species, were sampled (Table 1). Consistent with previous findings [14, 27], the distribution of these species was not homogeneous across the five study villages. More than one species was found in 4 of the 5 villages but they varied greatly in their relative abundance (Table 1). *Anopheles farauti* (*s.s*.) was predominant in the coastal villages whereas *An. punctulatus* (*s.s*.) was predominant in the inland villages. *Anopheles farauti* no. 4 was sampled only in Kokofine but at high abundance. *Anopheles koliensis* was sampled in low numbers at multiple sites. *Anopheles bancroftii* was sampled mostly in Mirap but was uncommon, and absent at most sites. *Anopheles longirostris* was sampled in low numbers in Mirap but was one of the two dominant species in Wasab. *Anopheles hinesorum* was found only in Matukar and Mirap in very low numbers.

**Table 1:** Number of sampled blood-engorged mosquitoes sorted according to their species and the village from which they were collected. Number in parenthesis represents the percent proportion of the corresponding species relative to the other species collected from a village

Mosquito species	Matukar *n* (%)	Mirap *n* (%)	Wasab *n* (%)	Dimer *n* (%)	Kokofine *n* (%)
*An. bancroftii*	0 (0)	66 (4.2)	3 (1.1)	1 (2.2)	0 (0)
*An. farauti* (*s.s*.)	55 (85.9)	1,443 (91.9)	20 (7.2)	2 (4.3)	0 (0)
*An. hinesorum*	3 (4.7)	5 (0.3)	0 (0)	0 (0)	0 (0)
*An. farauti* no. 4	0 (0)	0 (0)	0 (0)	0 (0)	483 (100)
*An. koliensis*	0 (0)	22 (1.4)	31 (11.2)	4 (8.7)	0 (0)
*An. longirostris*	2 (3.1)	19 (1.2)	99 (35.7)	4 (8.7)	0 (0)
*An. punctulatus* (*s.s*.)	4 (6.3)	15 (1)	124 (44.8)	35 (76.1)	0 (0)
Total	64 (100)	1,570 (100)	277 (100)	46 (100)	483 (100)

### Host selection

**Fig. 3:**
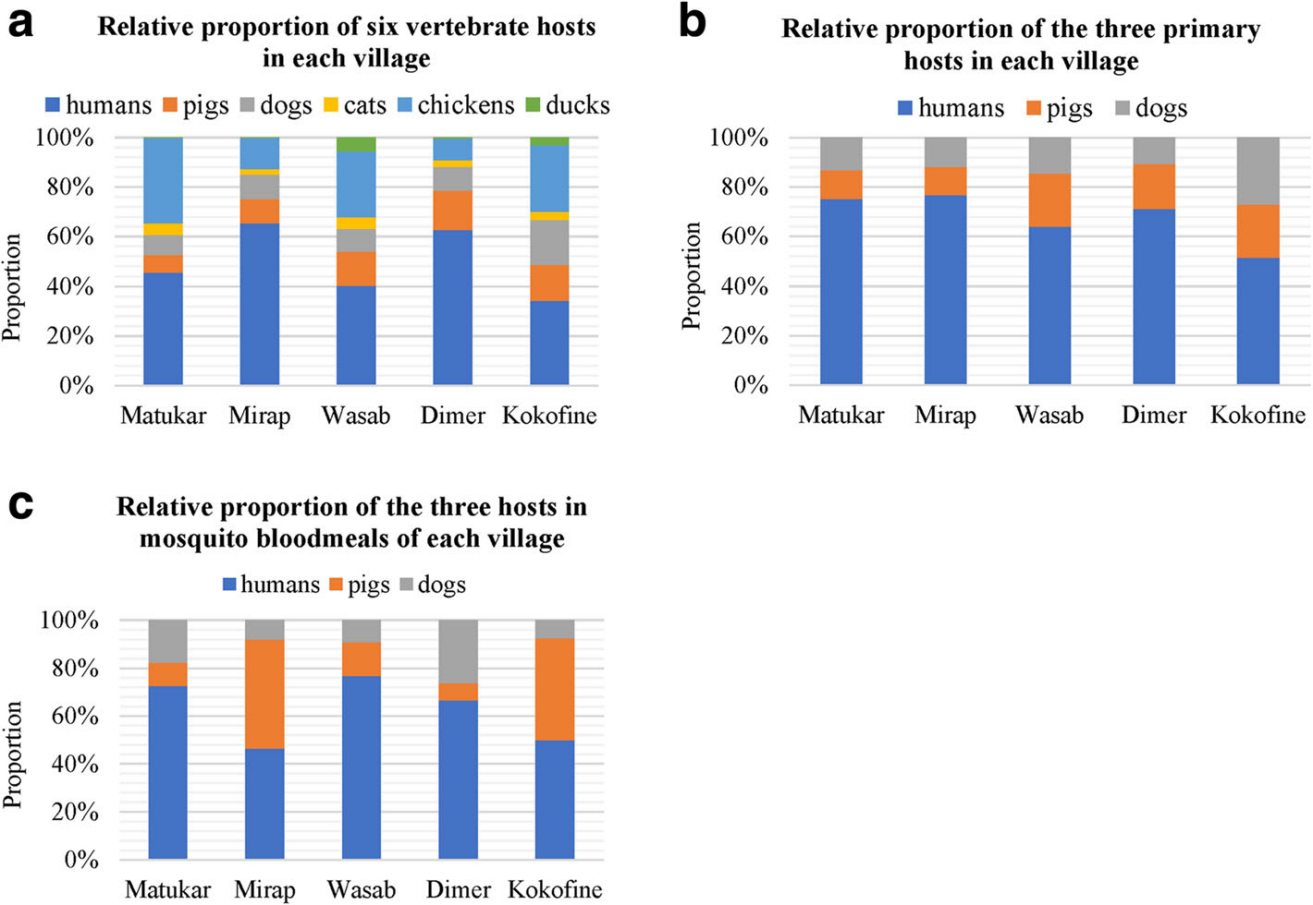
Bar charts showing the relative abundance of six vertebrate host species that were surveyed in Matukar (*n* = 950), Mirap (*n* = 1,121), Wasab (*n* = 523), Dimer (*n* = 992) and Kokofine (*n* = 488) village (**a**), the relative proportions of the three primary hosts in each village (Dimer: *n* = 874; Kokofine: *n* = 325; Matukar: *n* = 575; Mirap: *n* = 954; and Wasab: *n* = 330) (**b**) and the proportion of the three primary hosts in the mosquito blood meals for each village (Dimer: *n* = 42; Kokofine: *n* = 443; Matukar: *n* = 51; Mirap: *n* = 1,232; and Wasab: *n* = 220) (**c**)

Two thousand four hundred and twenty-two of the 2,440 anophelines (99.0%) yielded DNA for analysis. Of these, 2,142 (88.4%) were tested for source of host blood meal, and 2,061 (96.2%) yielded an interpretable gel phenotype or satisfactory BLAST search result for host source. Thus, 84.5% of the original, field-caught, blood-fed anophelines were successfully tested for host source. Eighty-six (4.2%) had mixed blood meals (44, human-pig; 30, human-dog; and 12, dog-pig). The remainder fed on a single host species. From our demographic surveys, there were six visually obvious vertebrate species (humans, pigs, dogs, cats, chickens and ducks) present in all of the villages (Fig. 3a). However, only humans, pigs, and dogs were found in the blood meals (Fig. 3c). The generic primers did not detect other mammal or avian species other than humans, dogs and pigs in the blood meals. The relative proportions of each of the three hosts by count is shown in Fig. 3b. Tests of the proportions of hosts available compared with the proportion actually utilized as reflected by blood meal analyses showed that for five of the six combinations of species and villages analyzed [*An. punctulatus* (*s.s*.) in Dimer; *An. punctulatus* (*s.s*.) in Wasab; *An. longirostris* in Wasab; *An. farauti* (*s.s*.) in Mirap; and *An. farauti* no. 4 in Kokofine], the relative proportion of the three hosts in the blood meals was not proportional to their relative proportion in the village (Table 2). Only *An. farauti* (*s.s*.) in Matukar showed a proportional association between host selection and host availability with this test, but the sample size of blood fed mosquitoes was modest (*n* = 43). For *An. hinesorum*, six fed on human, one fed on pig, and one on dog.

**Table 2:** Results for homogeneity of proportion (3 × 2 contingency table) test comparing the relative number of the three primary hosts in the village with their number in the mosquito blood meals. Mosquito feeding is considered disproportional to the host availability when the *χ* ^2^ test appeared statistically significant and proportional when insignificant

Village	*Anopheles* species	Host species	No. of hosts in the village	No. of hosts in the blood meals	*χ* ^2^ test statistic	*P*-value	Feeding outcome
Matukar	*An. farauti* (*s.s*.)	humans	432	33	*χ* ^2^ = 2.62	0.25	proportional
		pigs	67	2			
		dogs	76	8			
Mirap	*An. farauti* (*s.s*.)	humans	732	541	*χ* ^2^ = 281.9	0.0005	disproportional
		pigs	110	522			
		dogs	112	94			
Wasab	*An. punctulatus* (*s.s*.)	humans	211	76	*χ* ^2^ = 7.2	0.02	disproportional
		pigs	71	10			
		dogs	48	17			
Wasab	*An. longirostris*	humans	211	57	*χ* ^2^ = 8.7	0.015	disproportional
		pigs	71	14			
		dogs	48	2			
Dimer	*An. punctulatus* (*s.s*.)	humans	622	20	*χ* ^2^ = 11.26	0.009	disproportional
		pigs	158	2			
		dogs	94	9			
Kokofine	*An. farauti* no. 4	humans	167	221	*χ* ^2^ = 70.7	0.0005	disproportional
		pigs	70	189			
		dogs	88	33			

*Note*: degrees of freedom is irrelevant to report along with the Pearson’s *χ* ^2^ result because the *P*-value was computed by Monte Carlo simulation

**Fig. 4:**
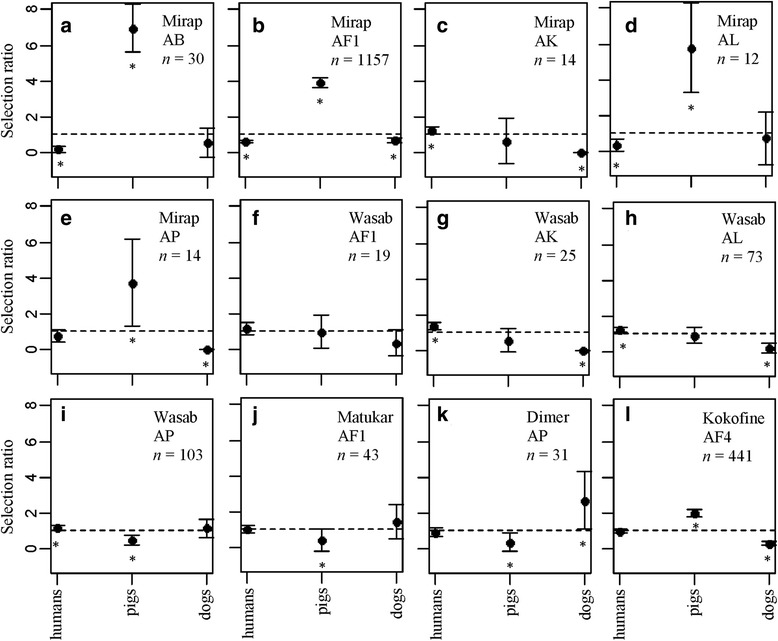
Graphs showing the Manly host selection ratio with 95% confidence interval bar for humans, pigs and dogs by five *Anopheles* species from Mirap (**a-e**) four species from Wasab (**f**-**i**) and one species each from Matukar (**j**), Dimer (**k**) and Kokofine (**l**). Mosquito species are abbreviated: AB, *An. bancroftii*; AF1, *An. farauti* (*s.s*.); AK, *An. koliensis*; AL, *An. longirostris*; AP, *An. punctulatus* (*s.s*.); AF4, *An. farauti* no. 4. The broken horizontal line marks the Manly ratio value 1. The asterisks indicate Manly ratio that are significantly greater than 1 (over-selection of the host species) or less than 1 (under-selection)

The Manly resource selection ratio revealed variation in host selection among different species within the same village and among different populations of the same species amongst villages. For Mirap, the most dominant species, *An. farauti* (*s.s*.), over-selected pigs compared to dogs and humans (Fig. 4b). The same was true for three of the other species in Mirap, i.e. *An. bancroftii*, *An. longirostris* and *An. punctulatus* (*s.s*.) (Fig. 4a, d and e), while *An. koliensis* over-selected humans (Fig. 4c) although sample size was modest (*n* = 14). For Wasab, *An. koliensis*, *An. longirostris* and *An. punctulatus* (*s.s*.) (Fig. 4g, h and i) over-selected humans, whereas *An. farauti* (*s.s*.) (Fig. 4f) selected hosts in proportion to their relative abundance. In Matukar, *An. farauti* (*s.s*.) under-selected pigs but selected humans and dogs in proportion to their relative abundance (Fig. 4j); and at Dimer, *An. punctulatus* (*s.s*.) over-selected dogs (Fig. 4k). In Kokofine, where sample size was generous (*n* = 441), *An. farauti* no. 4 over-selected pigs (Fig. 4l).

### Effect of LLIN usage on mosquito host selection

**Fig. 5:**
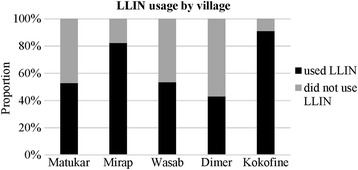
Bar graph showing the proportion of human population of each village that did and did not sleep under a bed net the night before the survey

LLIN usage varied among the study villages. Kokofine had the highest usage (91% of people used LLIN) followed by Mirap (82%), whilst usage was lower in Wasab (53.6%), Matukar (53%) and Dimer (43%) (Fig. 5). Logistic regression analysis showed that the odds of feeding on humans by *An. farauti* (*s.s*.) was statistically lower in Mirap compared to Matukar (odds ratio, OR = 0.25; 95% confidence interval (CI): 0.12–0.47; *P* < 0.001) or Wasab (OR = 3.04; 95% CI: 1.16–9.5; *P* = 0.03). Similarly, the odds of feeding on humans for this species was higher in Wasab compared to Mirap (OR = 3.04; 95% CI = 1.16–9.5; *P* = 0.03). The odds of feeding on humans by *An. longirostris* was higher in Wasab compared to Mirap (OR = 7.4; 95% CI: 2.26–27.1; *P* = 0.001). A weak statistical difference in the odds of feeding on humans by *An. punctulatus* (*s.s*.) for villages with high or low LLIN usage was observed. No statistical difference was observed for *An. koliensis* in Wasab versus Mirap. *Anopheles bancroftii* and *An. farauti* no. 4 were not tested as they were found in only a single village.

## Discussion

This study found that humans, pigs, and dogs were the primary hosts of malaria vectors in PNG. The narrow host range observed here is consistent with previous studies [10, 28–31], although some previous studies also detected a small number of mosquitoes that had fed on cats, chickens, opossums and bats. This difference may be due to the use of different mosquito sampling methods but those hosts were uncommon in all cases, compared to the three primary ones.

A number of studies [10, 28–31] have described the host preference of PNG vectors based on the patterns of host selection using human blood index (HBI), which is the proportion of mosquito blood meals obtained from human hosts [4, 32]. A mosquito species that had consistently high HBI was classified as anthropophilic (“human loving”), whilst those with consistently low HBI as zoophilic (“animal loving”); a variable HBI was considered opportunistic (i.e. selects hosts indiscriminately) in its host preference. However, the HBI does not take into account the relative abundance of the different host species within a mosquito’s foraging range. Various measures including the forage ratio [33], the feeding index [34], and the feeding preference index [35] expressed as the Manly resource selection ratio [36, 37], have been used to evaluate the mosquito host selection. These measures are inter-related and each requires some information of the range of available hosts, their relative abundance and related attributes. The Manly resource selection ratio was used in this study because it provides a statistical test for departure from randomness (i.e. equal to unity) with the 95% confidence interval.

A range of factors and processes influence mosquito host selection. These include host availability, host density, physical access to hosts, differential host attractiveness, behavior of hosts in response to mosquitoes’ attempts to feed, and a mosquito’s intrinsic host preference [1, 2]. The first part of this study emphasized relative abundance of hosts as a determining factor, and found that mosquito host selection was not merely a function of relative host abundance. Rather, strong biases in host selection towards pigs in Mirap and Kokofine and towards humans in Wasab, shown by both the test of proportions and the Manley host selection ratio, indicated that other factors impinged on it. One likely factor causing the observed host selection bias was unavailability of many human hosts due to protective effects of LLIN. The *Anopheles* mosquitoes of the different species within the same village would have been exposed to the same level of LLIN usage as well as other extrinsic factors. Thus, any differences among the species in their host selection can be attributed to variation in the biological factors, particularly their innate host preference. Therefore, the observed variation in the host selection among the five mosquito species within Mirap and four species within Wasab villages, where mosquito diversity was sufficient to compare interspecies host selection, supports the hypothesis that the vector species differ in their innate host preference. On the other hand, variation in the host selection of the same species among different villages can be attributed to the variation in LLIN usage and host availability among these villages.

The host preference of each *Anopheles* species was delineated by comparing their host selection in different villages with the level of LLIN usage. Three species, *An. farauti* (*s.s*.), *An. punctulatus* (*s.s*.) and *An. longirostris*, can be considered opportunistic as they over-selected pigs in Mirap, where LLIN usage was high, but over-selected humans or fed on the three hosts in proportion to their relative abundance in Wasab and Matukar, where the LLIN usage was low. That is, the opportunistic nature of their host preference allows them to respond plastically to varying LLIN usage. This was supported by logistic regression test which showed that the low anthropophagy exhibited by these species was associated with higher LLIN usage (although *An. punctulatus* (*s.s*.) was statistically weak). This assertion is consistent with previous studies, which found large variations in the HBI of *An. punctulatus* (*s.s*.), *An. farauti* (*s.s*.) and *An. longirostris* of various populations [28, 29]. Although *An. farauti* no. 4 was found in only one village and its host selection is reported here for the first time, marked over-selection of pigs indicates that it is also an opportunistic species, responding plastically to the high LLIN usage in Kokofine. In contrast to the opportunistic species, *An. koliensis* exhibited an anthropophilic host preference. It consistently over-selected humans in both Wasab and Mirap and logistic regression test showed no association between its high anthropophagy and low LLIN usage. This finding contrast with those of Charlwood et al. [29] and Burkot et al. [28] who found some populations of *An. koliensis* with relatively low HBI. However, these studies used morphological keys [19], now known to be unreliable [8, 38], to identify the different species in the *An. punctulatus* (*s.l*.) complex. Their use of morphological keys may have resulted in grouping of different species of varying host preference into the single taxon *An. koliensis*. Molecular based methods for species identification was used in this study to avoid this problem. The species *An. bancroftii* provides a strong contrast to *An. koliensis*, showing a highly zoophilic tendency. Its selection of human hosts was low even though humans were in higher abundance than pigs in Mirap. This finding is consistent with a previous study [30] which found *An. bancroftii* with very low HBI even when the other species in the same village had high HBI.

The modulation of host selection by bed nets has been observed both in PNG and elsewhere. A study in Kenya showed that the majority of blood meals taken by *Anopheles funestus* and *Culex quinquefasciatus* changed from humans before permethrin-impregnated bed nets was distributed to non-humans after the bed net distribution [39]. In a coastal village of Madang, PNG, the proportion of blood meals taken on humans by *An. farauti* (*s.l*.) dropped from 70% before permethrin-impregnated nets were distributed to 38% after the bed net distribution [40]. In the Wosera district of East Sepik province, PNG, increased use of insecticide-free bed nets resulted in a decline of the HBI of *An. punctulatus* (*s.s*.), but not of *An. koliensis* [31]. The persistently high HBI of *An. koliensis* in contrast to *An. punctulatus* (*s.s*.) despite the high bed net coverage in Wosera can be interpreted in terms of its strong preference for humans and inflexibility to utilize other hosts. This inflexibility can cause *An. koliensis* to pursue humans into the house, making it more vulnerable to the insecticidal effects of LLIN than the opportunistic species. Indeed, Hetzel et al. [13] and Reimer et al. [14] showed that while the population size of all *Anopheles* species was reduced after roll-out of LLIN in PNG, *An. koliensis* was affected the most in all their study sites.

The use of LLIN for controlling mosquito vectors remains the primary malaria intervention method in PNG. The reduction of malaria incidence, prevalence [13] and transmission intensity [14] in PNG in recent years have been attributed to the intensification of nationwide LLIN campaign over the last decade [15–17]. No resistance to pyrethroids has been detected in malaria vector populations in PNG [41, 42], but transmission continues to persist, perhaps in large part because of the host selection behavior of the opportunistic vector species described whose lack of dependence on human blood allows them to escape the lethal effect of LLIN. These vector species live in sympatry and co-transmit malaria in most of the endemic areas of PNG [12, 14, 27, 29–31, 43–46]. Therefore, while LLIN may affect the more anthropophilic and vulnerable species such as *An. koliensis*, transmission is still sustained by the more opportunistic and behaviorally plastic species. This condition, when combined with increased outdoor and early-evening biting observed in some vector population of PNG ([14] and unpublished data), presents a challenge to the LLIN program in PNG as well as the rest of the South West Pacific region where the opportunistic species *An. farauti* (*s.s*.) and *An. punctulatus* (*s.s*.) are the primary regional vectors.

This study was not without limitations. First, although the statistical analysis of host selection was based on the total number of the three primary hosts in the village, there were variations in the relative number of these hosts among households. Therefore, the household-level variation can bias the host selection results because mosquitoes were sampled near a household throughout the night. This bias was minimized by relocating each BS to a new location in the village every subsequent night to try and capture a good representation of the village. Secondly, the mobility of domestic hosts, including humans, throughout the night while mosquitoes were being sampled on the BS can change the actual availability of the hosts and bias the result of host selection. However, it was observed that although the animals roamed freely, dogs and pigs were found mostly close to their owner’s house during the night. Thirdly, readers may be concerned with bias associated with indoor resting mosquitoes not sampled by the BS method. The effect of this bias is minimal as members of the *An. punctulatus* (*s.l*.) group are primarily exophilic [47] even when they feed indoors.

## Conclusions

Except *An. koliensis* and *An. bancroftii*, which are anthropophilic and zoophilic respectively, the rest of the vector species are opportunistic blood feeders. The host selection plasticity of the opportunistic vectors of PNG can potentially limit the success of the LLIN program. While malaria elimination by the LLIN program is achievable from areas of PNG where *An. koliensis* is the only vector species present, it is difficult for LLIN alone to achieve malaria elimination from areas occupied by both *An. koliensis* and the opportunistic species. The opportunistic species will be resilient to the LLIN and continue to transmit the disease. Because *An. koliensis* is always found living in sympatry with the other species, reliance on LLIN alone is inadequate to achieve local malaria elimination. Alternative vector control methods or strategies need to be developed and implemented alongside the LLIN program in PNG.

## Additional file

Additional file 1:
**Table S1.** Dates of mosquito sampling in each village in the years 2012, 2013 and 2015. Shaded cells represent the dates in which mosquitoes were sampled. Color codes correspond to different villages. Gradient shades represent villages where mosquitoes were sampled simultaneously. (XLSX 13 kb)

## Abbreviations

BLAST: Basic local alignment search tool
BS: Barrier screen
CI: Confidence interval
DNA: Deoxyribonucleic acid
dNTP: Deoxyribonucleotide triphosphate
HBI: Human blood index
LLIN: Long-lasting insecticidal nets
OR: Odds ratio
PCR: Polymerase chain reaction
PNG: Papua New Guinea
*s.l*.: *sensu lato*
*s.s*.: *sensu stricto*
